# Understanding violence as a health determinant among ethnic communities in Colombia’s Pacific region: a qualitative analysis

**DOI:** 10.1590/0102-311XEN084925

**Published:** 2026-08-31

**Authors:** Anderson Rocha-Buelvas, Ana Paola Mogollón-Hernández

**Affiliations:** 1 Facultad de Ciencias de la Salud, Universidad de Nariño, Pasto, Colombia.

**Keywords:** Indigenous Peoples, Black People, Armed Conflict, Violence, Social Determinants of Health, Pueblos Indígenas, Población Negra, Conflicto Armado, Violencia, Determinantes Sociales de la Salud, Povos Indígenas, População Negra, Conflito Armado, Violência, Determinantes Sociais da Saúde

## Abstract

This study sought to understand the manifestations of violence affecting Indigenous and Afro-descendant communities in the Nariño Pacific region, Colombia, through the framework of the social determinants of health. A qualitative research design was implemented with 60 purposively selected participants, ensuring variation by ethnicity (49 Afro-descendant and 11 Indigenous), gender (34 women and 26 men), and role (institutional and community). All participants were adults affected by the armed conflict and registered in the National Victims Registry (RUV). Data were collected through in-depth interviews and the analysis of paradigmatic cases, complemented by field notes documenting contextual aspects of the data collection process. An inductive thematic analysis was conducted using the social determinants of health as the analytical framework. The findings reveal that violence affecting communities in the Nariño Pacific region operates as a structural determinant of health, characterized by systemic, symbolic, and historical configurations embedded within multiple social determinants (sociocultural, economic, political, environmental, and health service-related). These dynamics are reinforced by persistent symbols of domination, structural institutional crises, restricted access to fundamental rights, mistrust in the State, corruption, and the territorial control exercised by armed groups in these collective ethnic territories.

## Abstract (es)

Este estudio tuvo como objetivo comprender las manifestaciones de violencia que afectan a comunidades indígenas y afrodescendientes en la región del Pacífico de Nariño, Colombia, a través del marco de los determinantes sociales de la salud. Se implementó un diseño de investigación cualitativa con 60 participantes seleccionados intencionalmente, lo que garantizó una variación por etnia (49 afrodescendientes y 11 indígenas), género (34 mujeres y 26 hombres) y rol (institucional y comunitario). Todos los participantes eran adultos afectados por el conflicto armado y estaban registrados en el Registro Único de Víctimas (RUV). Se recopilaron los datos a través de entrevistas en profundidad y análisis de casos paradigmáticos, complementados con notas de campo que documentaron aspectos contextuales del proceso de recopilación de datos. Se realizó un análisis temático inductivo utilizando los determinantes sociales de la salud como marco analítico. Los hallazgos demuestran que la violencia que afecta a las comunidades de la región del Pacífico de Nariño funciona como un determinante estructural de la salud, que se caracteriza por configuraciones sistémicas, simbólicas e históricas insertas en diversos determinantes sociales (socioculturales, económicos, políticos, ambientales y relacionados con los servicios de salud). Estas dinámicas se ven reforzadas por símbolos persistentes de dominación, crisis institucionales estructurales, acceso restringido a derechos fundamentales, desconfianza en el Estado, corrupción y el control territorial ejercido por grupos armados en estos territorios étnicos colectivos.

## Abstract (pt)

Este estudo buscou compreender as manifestações da violência que afetam comunidades indígenas e afrodescendentes na região do Pacífico de Nariño, Colômbia, por meio do quadro dos determinantes sociais da saúde. Um desenho de pesquisa qualitativa foi implementado com 60 participantes selecionados propositalmente, garantindo variação por etnia (49 afrodescendentes e 11 indígenas), gênero (34 mulheres e 26 homens) e papel (institucional e comunitário). Todos os participantes eram adultos afetados pelo conflito armado e registrados no Registro Nacional de Vítimas (RUV). Os dados foram coletados por meio de entrevistas aprofundadas e análise de casos paradigmáticos, complementadas por notas de campo que documentaram aspectos contextuais do processo de coleta de dados. Foi realizada uma análise temática indutiva utilizando os determinantes sociais da saúde como estrutura analítica. Os achados revelam que a violência que afeta comunidades na região do Pacífico de Nariño opera como um determinante estrutural da saúde, caracterizado por configurações sistêmicas, simbólicas e históricas embutidas em múltiplos determinantes sociais (socioculturais, econômicos, políticos, ambientais e relacionados aos serviços de saúde). Essas dinâmicas são reforçadas por símbolos persistentes de dominação, crises institucionais estruturais, acesso restrito a direitos fundamentais, desconfiança no Estado, corrupção e o controle territorial exercido por grupos armados nesses territórios étnicos coletivos.

## Introduction

Violence is not an isolated event; it constitutes a multidimensional process encompassing direct, structural, institutional, and symbolic phenomena that reorganize social life and shape the healthcare trajectories of individuals and communities ^1^ ^,^ ^2^. It operates through mechanisms that inflict harm, exert influence, and shape the behaviors of individuals and groups, with effects that extend to the social determinants of health (SDH) - those social systems that structure and regulate access to both health resources and health risks ^3^. Several studies suggest that individual determinants, such as early exposure to trauma, disruptive behavior, and impulsivity, are linked to more aggressive forms of interpersonal violence, whereas socio-contextual determinants, encompassing social, political, and cultural factors, are associated with the use of violence in contexts of war ^4^ ^,^ ^5^ ^,^ ^6^.

From the perspective of the SDH, institutional actions, public policies, resources, and environmental risks that shape the contexts in which people are born, grow, work, and age generate mechanisms that expose populations to multiple forms of violence and their effects on physical and mental health (e.g., psychosocial distress, somatic symptoms, chronic diseases). These mechanisms also contribute to inequities in the utilization of institutional services at the political, social, and health levels, manifested in social inequality, delays in care, preventable adverse events, deficient health infrastructure, and ethno-racial discrimination ^7^ ^,^ ^8^ ^,^ ^9^. Thus, the explanatory framework of the SDH enables us to see violence not as an isolated event, but as a public health issue produced and reproduced by the system itself ^1^ ^,^ ^10^.

In Colombia, violence associated with the armed conflict is characterized by its multidimensional and territorial nature, combining direct attacks (such as armed confrontations, the use of landmines, and other explosives) with mechanisms of social control and territorial dispute, including displacement, confinement, and restrictions on mobility and access to public services. This dynamic unfolds within a context of social fragmentation and environments marked by poverty, inequality, and State fragility, factors recognized by the health sector as social determinants that exacerbate intrafamilial, interpersonal, and intimate partner violence, thereby worsening health outcomes ^7^ ^,^ ^11^ ^,^ ^12^. This is the case in the Department of Nariño, Colombia, which, in 2024, reported the highest national levels of displacement and mobility restrictions resulting from the armed conflict. A report by the International Committee of the Red Cross (ICRC) highlighted the worsening humanitarian deterioration in the region, marked by deepening poverty and inequality associated with the sharp increase in victims of the conflict ^13^.

From this perspective, life in the Nariño Pacific region, located in the southeast of Colombia, unfolds amid the coexistence of direct violence (physical acts of aggression), structural violence (social, economic, and political inequalities embedded within societal structures), and cultural violence (beliefs, norms, and practices that legitimize oppression and discrimination) ^2^ ^,^ ^14^. These forms of violence have produced bodies in a constant state of emergency - physically and psychologically affected by material, symbolic, cognitive, and emotional forces that perpetuate the chronicity of both physical and mental harm ^15^. As of February 2024, this territory had recorded a total of 434,249 victims of the armed conflict, distributed by municipalities in the Nariño Pacific as follows: Barbacoas, 44,315; El Charco, 51,059; Francisco Pizarro, 7,427; La Tola, 10,414; Magüí Payán, 18,860; Mosquera, 7,335; Olaya Herrera, 32,364; Ricaurte, 18,201; Roberto Payán, 26,042; Santa Bárbara, 15,531; and Tumaco with 202,701, representing the municipality in the Nariño Pacific with the highest number of recorded victims ^16^.

According to ACAPS ^17^, this region of the Nariño Pacific maintains power relations established within a framework of social, cultural, political, and economic domination that creates structures of marginalization and exclusion for certain social groups. This has contributed to the intensification of violence and armed conflict, resulting in deaths, disease, and a decline in the quality of life among inhabitants, particularly within Afro-descendant and Indigenous communities, as armed groups entered these territories to impose rules that ultimately fractured social bonds ^14^.

In this context, a direct association between violence and adverse health outcomes is evident from the perspective of the SDH. However, an exploratory review conducted between 2012 and 2022 in PubMed, LILACS, and SciELO, using descriptors related to violence, social determinants, health, the Colombian Pacific, Indigenous peoples, and Afro-descendant communities, revealed no studies that directly analyze the various manifestations of violence affecting Indigenous and Afro-descendant populations in the Nariño Pacific through the explanatory framework of the SDH. Such analyses are essential to identify focal points for interventions aimed at reducing harm associated with violence. This gap therefore justified the qualitative approach adopted in the present study, which aimed to understand violence against Indigenous and Afro-descendant communities in the Nariño Pacific through the explanatory framework of the SDH, based on participants’ experiences, beliefs, and behaviors in relation to violence across 11 municipalities in the Nariño Pacific region of Colombia, territories characterized by extensive areas of collective land ownership belonging to Afro-descendant community councils and Indigenous peoples.

## Method

This qualitative study aimed to understand the manifestations and meanings of violence experienced by ethnic communities in the Nariño Pacific region of Colombia. A purposive sampling strategy was employed, with inclusion criteria based on ethnic affiliation (Afro-descendant or Indigenous), gender, age over 18 years, participant role (institutional or community-based), and verified status as a victim of the armed conflict, confirmed through the National Victims Registry (RUV - *Registro Único de Víctimas*), with participants’ authorization.

The study was reviewed and approved by the Research Ethics Committee of the University of Nariño (Approval Act n. 073, corresponding to project code 2794, approved on May 19, 2023). Subsequently, eligible individuals were identified, and informed consent was obtained. In accordance with national and international ethical standards for research involving human participants, confidentiality, anonymity, and respect for participants’ rights were guaranteed. Participants were also informed of their right to withdraw from the study at any stage of the research process.

Participant recruitment was conducted through engagement with State and non-State institutions, national and international foundations, as well as groups of social and community leaders and agents. For ethical, confidentiality, and security reasons, the specific names of these entities are not disclosed. This decision was guided by the principle of non-maleficence and informed by a territorial-level risk assessment.

The final sample consisted of 60 individuals who met the inclusion criteria: 49 persons belonging to Afro-descendant communities and 11 to Indigenous peoples. Of these, 34 were women and 26 were men, all adult victims of the armed conflict. The sample reflected diversity in terms of sexual orientation and gender identity, including four lesbian women, one transgender woman, and two gay men. Participants were distributed across 11 municipalities in the Nariño Pacific region: San Andrés de Tumaco (38 participants), Francisco Pizarro (3), Magüí Payán (3), Roberto Payán (3), Ricaurte (3), Mosquera (2), Olaya Herrera (2), Barbacoas (2), El Charco (2), La Tola (1), and Santa Bárbara de Iscuandé (1).

Data collection was conducted from May 22 to October 14, 2023, after ethical approval had been obtained. This process included in-depth interviews, which were audio-recorded with participants' prior consent and had an average duration of approximately 90 minutes. The interviews were complemented by field notes documenting contextual and interactional aspects of the data collection process.

An inductive thematic data analysis was conducted following the approach proposed by Braun & Clarke ^18^, involving a coding process aimed at identifying meaningful units of information, guided by the principles of qualitative coding described by Saldaña ^19^. Recurrent patterns and variations identified across interviews were grouped into themes and subthemes, generating provisional analytical categories. A subsequent reclassification process enabled the organization and consolidation of these categories, providing interpretive meaning to the data for understanding violence. The findings were systematized in matrices and accompanied by comprehensive descriptions for each theme and subtheme. Finally, the results were shared with participants to verify data credibility. The validity and reliability of this study are grounded in the qualitative research principles of credibility, transferability, and dependability ^20^ ^,^ ^21^.

The study population was classified by sociodemographic characteristics (Box 1), population group and ethnic identity (Table 1), and institutional actors (Box 2).

**Box 1:** Sociodemographic characterization of ethnic participants.

ETHNICITY: AFRO-DESCENDANT POPULATION				
ID	GENDER	AGE	MUNICIPALITY	PARTICIPANT ROLE
E1	Woman	58	San Andrés de Tumaco	Community - Community leader
E2	Woman	35	San Andrés de Tumaco	Institutional - Administrative
E3	Woman	65	San Andrés de Tumaco	Community - Community leader
E4	Woman	53	San Andrés de Tumaco	Institutional - Administrative
E5	Woman	57	San Andrés de Tumaco	Institutional - Administrative
E6	Man	51	San Andrés de Tumaco	Institutional - Administrative
E7	Woman	67	San Andrés de Tumaco	Community - Community leader
E8	Transgender woman	25	San Andrés de Tumaco	Institutional - Beneficiary
E10	Woman	68	San Andrés de Tumaco	Community - Social leader
E11	Woman	43	San Andrés de Tumaco	Institutional - Director/Manager
E12	Man	49	San Andrés de Tumaco	Institutional - Administrative
E13	Woman	62	San Andrés de Tumaco	Community - Community resident
E14	Woman	19	San Andrés de Tumaco	Institutional - Beneficiary
E15	Woman	41	San Andrés de Tumaco	Institutional - Administrative
E16	Woman	28	San Andrés de Tumaco	Institutional - Administrative
E17	Woman	33	San Andrés de Tumaco	Community - Community resident
E18	Man	58	San Andrés de Tumaco	Community - Social leader
E19	Woman	20	San Andrés de Tumaco	Institutional - Beneficiary
E20	Woman	53	San Andrés de Tumaco	Institutional - Administrative
E21	Woman	47	San Andrés de Tumaco	Institutional - Director/Manager
E22	Woman	24	San Andrés de Tumaco	Institutional - Beneficiary
E23	Woman	28	San Andrés de Tumaco	Institutional - Beneficiary
E24	Woman	21	San Andrés de Tumaco	Community - Community leader
E25	Man	20	San Andrés de Tumaco	Community - Community resident
E26	Man	41	San Andrés de Tumaco	Institutional - Administrative
E28	Woman	19	San Andrés de Tumaco	Community - Community resident
E29	Man	26	San Andrés de Tumaco	Institutional - Administrative
E30	Man	60	San Andrés de Tumaco	Community - Community leader
E31	Man	25	San Andrés de Tumaco	Institutional - Administrative
E32	Man	43	San Andrés de Tumaco	Institutional - Administrative
E33	Man	52	San Andrés de Tumaco	Institutional - Director/Manager
E34	Man	46	San Andrés de Tumaco	Community - Social leader
E39	Woman	24	Francisco Pizarro	Institutional - Beneficiary
E40	Woman	42	Francisco Pizarro	Community - Social leader
E41	Man	27	Francisco Pizarro	Community - Community leader
E42	Woman	26	Magüí Payán	Institutional - Beneficiary
E43	Woman	29	Magüí Payán	Community - Community resident
E44	Man	30	Magüí Payán	Community - Community resident
E45	Woman	22	Roberto Payán	Institutional - Beneficiary
E47	Man	29	Roberto Payán	Community - Community resident
E48	Woman	66	Mosquera	Community - Social leader
E49	Man	33	Mosquera	Community - Community resident
E50	Woman	27	Olaya Herrera	Community - Community leader
E51	Man	60	Olaya Herrera	Institutional - Director/Manager
E53	Man	31	Barbacoas	Community - Community resident
E54	Woman	51	El Charco	Community - Community leader
E55	Man	54	El Charco	Community - Community resident
E58	Woman	19	La Tola	Community - Community resident
E60	Man	64	Santa Bárbara de Iscuandé	Community - Community leader
ETHNICITY: INDIGENOUS POPULATION				
ID	GENDER	AGE	MUNICIPALITY	PARTICIPANT ROLE
E9	Woman	54	San Andrés de Tumaco	Institutional - Beneficiary
E27	Man	39	San Andrés de Tumaco	Institutional - Administrative
E35	Man	27	San Andrés de Tumaco	Institutional - Beneficiary
E36	Man	25	San Andrés de Tumaco	Institutional - Administrative
E37	Man	28	San Andrés de Tumaco	Institutional - Beneficiary
E38	Man	67	San Andrés de Tumaco	Community - Social leader
E46	Man	64	Roberto Payán	Community - Social leader
E52	Woman	38	Barbacoas	Institutional - Director/Manager
E56	Woman	45	Ricaurte	Community - Community resident
E57	Man	56	Ricaurte	Institutional - Administrative
E59	Woman	62	Ricaurte	Institutional - Beneficiary

**Table 1:** Segmentation by population group and ethnic identity.

Population group/Role	Afro-descendant	Indigenous
Institutional		
Directors/Managers	4	1
Administrative staff	12	3
Beneficiaries	8	4
Community		
Social leaders	5	2
Community leaders	9	0
Community residents	11	1
Total (N = 60)	49	11

**Box 2:** Characterization of institutional actors.

#	LEGAL NATURE	SCOPE	PRIMARY ROLE
1	State institution	Municipal	Provision of health services
2	State institution	National	Assistance to victims
3	State institution	National	Search, clarification, and family support
4	State institution	Subnational	Health authority, public health, and surveillance
5	International NGO	International/National	Implementation in mental health, protection, and access to justice
6	Trade union organization	Regional	Professional representation and rights advocacy
7	State institution	Municipal	Access to justice, conciliation, and alternative dispute resolution (ADR)
8	Local foundation	Municipal	Community/Protection programs
9	Non-State ethnic organization	Regional (Nariño/Cauca)	Community governance and rights defense
10	Intergovernmental organization	International	Multilateral cooperation and human rights
11	International NGO	International/National	Child protection, education, and gender initiatives

NGO: nongovernmental organization.

## Results

The analysis of violence in the Nariño Pacific against Indigenous and Afro-descendant communities draws on the typologies of violence described by Galtung ^2^ and the explanatory framework of the SDH proposed by Farmer ^1^ and Marmot ^10^. The findings reveal consistent patterns of civic coexistence under a coercive economy, marked by symbols of armed power and restrictions of rights that function as structural determinants of violence across the municipalities of San Andrés de Tumaco, Francisco Pizarro, Magüí Payán, Roberto Payán, Mosquera, Olaya Herrera, Barbacoas, El Charco, Ricaurte, La Tola, and Santa Bárbara de Iscuandé. To preserve confidentiality, participants’ testimonies are identified using the codes “E1” through “E60”, reflecting the recurrent patterns observed during the research process and organized under the analytical category (Box 3).

**Box 3:** Organizational logic of violence against ethnic communities in Colombia’s Nariño Pacific.

THEME/SUBTHEME	BRIEF DEFINITION	CATEGORIES
Direct expressions		
Resource acquisition for war	Activities that finance the armed conflict	Illicit crops; drug trafficking; kidnapping for ransom; extortion; payment of “*vacunas*” (protection fees)
Attacks on the medical mission	Aggressions against health personnel/means within the conflict	Killings of health workers; threats; kidnapping; attacks on medical transport
Modalities of violence	Immediate forms in which harm is manifested	Physical and verbal assaults; killings; threats; massacres; terrorist acts; attacks on property; kidnapping; torture; confinement; armed confrontation; enforced disappearance; forced displacement; sexual violence; antipersonnel mines; illicit recruitment
Cultural violence		
Cognitive frame of violence	Thoughts, emotions, and perceptions linked to violence	Intrusive memories; distrust; fear; frustration; helplessness; risk perception; anger; resentment; desires for aggression, justice, and revenge
Ideological frame of violence	System of ideas that legitimizes/normalizes violence	Idealization of armed groups; indoctrination into coercive power; asymmetric power; dehumanization of the other; overvaluation of power and money
Symbolic frame of violence	Symbols that represent violence.	Firearms; speedboats with 200 HP engines; specific neighborhoods; rural zones (*veredas*); Immediate Attention Centers (CAI) of the Colombian National Police; coca leaf (“*mata que mata*”, i.e., “the plant that kills”)
Structural violence		
Survival needs	Lack of access to basic means of living	Difficulties accessing food
Well-being needs	Violations of quality of life and services	Barriers to education; barriers to employment; State inefficacy/abandonment; negligence by authorities; lack of guarantees for public functions; poor access to the health system; negligence in health/protection; inadequate housing
Freedom needs	Restrictions on interaction and rights	Restrictions on claiming rights; limits on assembly; coercion of free expression; coercion of free mobility
Identity needs	Violations of personal/cultural recognition	Stigmatization; discrimination; denial of rights guarantees

### Direct expressions of violence

The findings show that direct violence is primarily expressed through systematic mechanisms of armed control, aimed both at securing resources for war and at the exercise of parallel forms of governance by non-State armed actors over territories and populations. These mechanisms are not limited to the use of force; they also operate through the institutionalization of rules, sanctions, and practices of social regulation that structure the everyday life of these communities. As participants described:

E15: “*Nadie quiere invertir porque es que el que quiere medio colocar un negocio ya le están pidiendo vacuna*” [No one wants to invest, because anyone who tries to set up even a small business is immediately asked to pay extortion].

E48: “*Hablar de vacunar o extorsionar es cuando los grupos armados van hasta tu negocio y te exigen que un porcentaje o una cuota diaria o semanal o mensual*” [Talking about vaccination or extortion refers to when armed groups come to your business and demand a percentage or a daily, weekly, or monthly fee].

Extortion of the formal commercial sector has thus become a routine practice that functions as a structural mechanism of coercive appropriation of resources, through which armed actors capture economic surplus from a range of productive activities. This extortion regime discourages investment and restricts access to formal employment, weakens legal markets, and consolidates economic dynamics based on informality and illegality. From this perspective, direct violence operates as a mechanism articulating structural vulnerability, by conditioning opportunities for work, income, and well-being, and by deepening the historical inequalities affecting Indigenous and Afro-descendant populations in these territories.

In addition, serious violations of International Humanitarian Law and of the principle of medical neutrality were identified through systematic attacks against the medical mission, which function as mechanisms of territorial control that condition the operation of the health system and the access to care. These include restrictions on ambulance transit, intimidation of health personnel, and intermittent closure of health services. In this context, healthcare delivery becomes exposed to risk, undermining continuity of care and service capacity. Consequently, disruptions in timely access to emergency care, antenatal check-ups, continuous treatments, and specialized services increase the burden of disease, particularly in dispersed rural areas.

At the same time, the attrition of health personnel, driven by direct threats, insecurity, and emotional exhaustion, contributes to a reduced effective supply of services, overburdened work teams, and deterioration in the quality of care. These findings clearly demonstrate that armed violence directly interferes with the realization of the right to health, as reflected in participants’ accounts:

E3: “*No dejan pasar las ambulancias*” [They do not allow ambulances to pass].

E10: “*Llegan a rematarlos al hospital y hasta los médicos o enfermeras han salido heridos*” [They come to finish them off at the hospital, and even doctors and nurses have been injured].

The persistent presence of homicides, enforced disappearances, torture, anti-personnel landmines, and severe restrictions on mobility configures an environment of permanent threat, in which everyday life unfolds under constant conditions of risk and uncertainty. Beyond constituting expressions of direct violence, these acts also function as devices of social control that regulate behavior, restrict circulation, and shape community interactions, influencing decisions related to work, education, healthcare seeking, and community participation:

E12: “*A cualquiera lo matan*” [Anyone can be killed].

E18: “*Se presentan desapariciones forzadas, tortura, minas antipersona que dejan amputaciones, el impedimento de moverse a cualquier lado*” [There are forced disappearances, torture, and anti-personnel landmines that cause amputations, as well as restrictions on moving freely].

The breakdown of support networks, social isolation, and the inability to organize collective responses to risk further increase community vulnerability, particularly among children, women, older adults, and community leaders. In this regard, violence affects populations differentially according to gender and life course, producing a clear stratification of risk: children face the risk of recruitment and early trauma; adolescents and young people are subject to co-optation and criminalization; women experience sexual and economic violence; and men face selective persecution, extortion, and homicide. As participants noted:

E11: “*No se puede hablar con nadie*” [You can’t talk to anyone].

E50: “*Solo cuando vienen los helicópteros o se escuchan lanchas motor 200*” [Only when helicopters arrive or when the sound of 200-horsepower motorboats is heard].

E60: “*En el territorio hay caciques, grupos y esto pone en riesgo a todas las poblaciones*” [There are local power brokers and armed groups in the territory, and this puts all populations at risk].

### Expressions of cultural and structural violence

The testimonies show how the constant fear of retaliation by non-State armed groups restricts communication among neighbors, fosters silence and self-censorship, and erodes interpersonal trust, all of which are fundamental elements for collective action and community governance. In this context, everyday practices such as talking, organizing, or moving freely acquire a heightened sense of risk, leading to the disarticulation of community-based mechanisms of care, mutual support, and collective problem-solving:

E23: “*No se puede hablar con nadie, si lo ven conversando con el vecino, creen que informa*” [You can’t talk to anyone; if they see you talking to a neighbor, they think you are informing on them].

Simultaneously, several accounts attribute power, status, and symbolic legitimacy to armed groups, establishing them as figures of authority and social reference points that exert particular influence on adolescents and young people, for whom these structures are perceived as viable life projects. Within this framework, the symbolic construction of armed power becomes an effective form of authority, protection, and social mobility:

E14: “*Ellos tienen el poder; con un arma en la mano hacen lo que quieren*” [They have the power; with a gun in their hand, they do whatever they want].

E31: “*Para algunos jóvenes, ‘meterse’ es el trofeo*” [For some young people, “joining them” is the trophy].

In this sense, cultural violence symbolically legitimizes armed domination by naturalizing its presence and authority within the territory, producing forms of social adaptation that, while oriented toward survival, contribute to the internalization of logics of domination, normalizing the use of force as a legitimate mechanism for social regulation and conflict resolution.

The findings further show that the imposition of norms, symbols, and practices alien to Indigenous and Afro-descendant worldviews has led to processes of forced acculturation that directly affect systems of ancestral knowledge, particularly those related to the medicinal use of plants, collective care practices, and food sovereignty. Thus, the transformation of coca, historically associated with cultural, ritual, and therapeutic uses, into an illicit monoculture imposed by armed actors is perceived by these populations as a tangible manifestation of the impact of violence on their territories. The expression “*la mata que mata*” symbolically conveys the loss of coca’s cultural and healing value, as well as its resignification as a driver of war and of the economy of violence:

E5: “*La mata que mata*” [The plant that kills].

This transformation not only alters the local productive structure but also redefines the relationship between community, nature, and health, displacing care practices grounded in ancestral knowledge with extractive dynamics that degrade the environment, contaminate ecosystems, and generate economic dependency. From this perspective, cultural and structural violence converge in the production of environmental, social, and health-related harms, by eroding the symbolic and material foundations that previously sustained the reproduction of life in these territories:

E28: “*Las lanchas ‘200’ dan miedo, porque cuando suena el motor, los niños se esconden*” [The “200” boats are frightening, because when the engine is heard, children hide].

Likewise, structural violence is persistently expressed through the progressive precarization of living conditions among Indigenous and Afro-descendant communities in the Nariño Pacific, manifested in the sustained loss of purchasing power, rising food prices, and increasing food insecurity. The findings indicate that these conditions are not the result of isolated economic dynamics, but rather of structural processes linked to armed territorial control, recurrent closures of fluvial and land routes, and the imposition of illicit economies that reconfigure supply chains. Rising prices of basic food items become a daily experience, forcing households to reduce the quantity, quality, and diversity of their diets, thereby directly affecting food and nutritional security.

This structural precarization has direct effects on population health, increasing the risk of malnutrition, chronic diseases associated with poor diets, worsening maternal and child health outcomes, and mental health impacts derived from economic stress and food-related uncertainty. Women, children, older adults, and dispersed rural households experience these dynamics more intensely, thereby deepening health inequities across the territory:

E9: “*Con 20 mil antes comíamos todo el día; ahora no alcanza ni para el desayuno*” [With 20,000 we used to eat all day; now it’s not even enough for breakfast].

E26: “*La vía cerrada sube la gasolina y no entra comida*” [The closed road raises the price of gasoline, and food doesn’t come in].

E37: “*El monocultivo ilícito trajo pobreza*” [The illicit monoculture brought poverty].

### Violence from the explanatory framework of the social determinants of health

This organizational logic of violence has jeopardized the guarantee of human rights, revealing environments that negatively impact the health of Indigenous and Afro-descendant peoples in the Nariño Pacific region of Colombia. From this organizational logic, and according to the report of the Commission on Social Determinants of Health of the World Health Organization ^21^, chaired by Michael Marmot ^10^, and the effects of power described by Farmer ^1^, violence was identified as a symptom of failed policies and inequities in living conditions, access to basic resources, and participation in society. Thus, violence is understood within an explanatory framework grounded in the SDH (Box 4).

**Box 4:** Explanatory framework of violence from the lens of social determinants of health in ethnic communities of Colombia’s Nariño Pacific.

THEME	SOCIAL DETERMINANTS OF HEALTH
Economic	Business absence and/or loss
	Changes in the productive structure
	Loss of productivity
	Household impoverishment
	Rising cost of living
	Illegal economy
Sociocultural	Rupture of the social fabric
	Modification of social relations
	Loss of ancestral knowledge
	Loss of customs
	Loss of cultural identity
	Loss of food sovereignty
	Erosion of values of respect and solidarity
	Weakening of community self-management processes
Political	Corruption
	Limitations on officials’ ability to perform their duties
	Reduced effectiveness of local governments
	Fear of political participation
	Weakening of local democracy
	Lack of guarantees for political participation
	Political control by armed groups
Environmental	Water, air, and soil contamination from war-related activities
	Water, air, and soil contamination due to chemical use
	Loss of biodiversity
	Inadequate waste management
Health services	Lack of guarantees for retention of health personnel
	Absence of permanently available specialized personnel
	Insufficient capacity to serve the population
	Geographic access barriers to health care
	Limitations in access to diagnostic and treatment technologies
	Limited supply of health services
	Limited access to medicines
	Weak health inspection and surveillance
Road infrastructure	Mobility impeded by road destruction or damage
	Mobility impeded by road closures
	Mobility limited by lack of adequate roadworks
	Mobility limited by transport costs
Public services	Interruption of electricity services
	Interruption of water services
	Lack of access to safe drinking water
	Limited access to public utilities
	Lack of investment to improve public services

The findings show that armed coercion imposes an economic model grounded in illegality that conditions community livelihood strategies. The population is compelled to participate in illicit economies, particularly coca cultivation, which emerges as a forced response to contexts of threat, territorial control, and the absence of safe productive alternatives. Participants’ accounts indicate that this imposition not only redefines economic activities but also reconfigures notions of work, stability, and future prospects, progressively eroding the licit economy:

E1, E6, E10, E13, E29, E54, E57: “*Hay que sembrar lo que ellos dicen*” [You have to plant what they tell you to].

E19: “*Al adquirir una cultura ilícita frente a una economía lícita se destruye una mentalidad de economía estable y legal*” [By adopting an illicit culture in place of a licit economy, a stable and legal economic mindset is destroyed].

Within this scenario, formal markets are undermined, the local productive fabric is weakened, and households are exposed to unstable incomes, indebtedness, and heightened economic vulnerability. From an SDH perspective, these conditions translate into food insecurity, chronic stress, and the precarization of everyday life, with both direct and indirect effects on physical and mental health.

At the sociocultural level, the results indicate that violence has generated deep fractures in family and community cohesion systems. Homicides, forced displacement, recruitment of children and adolescents, and involvement in illicit economies significantly alters the community dynamics by disintegrating family units and by reconfiguring everyday norms, social roles, and traditional forms of collective organization and care:

E14: “*Desde que llegó el conflicto armado lamentablemente vemos cómo los núcleos familiares se han destruido prácticamente al tener al hijo involucrado en estos grupos, cuando existe homicidios de los jefes de hogar*” [Since the armed conflict arrived, unfortunately we have seen how family units have been practically destroyed, when a son becomes involved in these groups, and when heads of household are killed].

Moreover, the intergenerational transmission of ancestral knowledge, values, and practices, associated with the worldviews of Indigenous and Afro-descendant communities and expressed through customs, rituals, and bonds of trust that sustain social cohesion and collective identity, has been profoundly disrupted by experiences and narratives linked to the armed conflict. These accounts reveal processes of community fragmentation and the loss of spaces for socialization associated with care, reciprocity, and coexistence:

E58: “*Costumbres muy bonitas que el conflicto ha roto...*” [Very beautiful customs that the conflict has broken...].

These processes give rise to forms of collective suffering that transcend individual distress, characterized by unresolved grief, enforced silence, and the loss of community spaces for emotional containment. At the same time, the weakening of social support networks reduces communities’ capacity to provide accompaniment, protection, and mutual care, elements that are fundamental for health in contexts of high vulnerability. Likewise, the loss of cultural and community referents increases exposure to substance use and risk behaviors, particularly among adolescents and young people, for whom the rupture of protective bonds and culturally meaningful future horizons intensifies psychosocial distress.

In the political sphere, the findings show that violence is sustained within a context of State fragility, crises of institutional legitimacy, normalization of corruption, and coercion of political participation, which limits the capacity of both communities and local governments to demand and guarantee rights aimed at responding to population needs:

E16: “*Estamos hablando también de un Estado corrupto*” [We are also talking about a corrupt State].

The accounts reveal a persistent fear of political participation, associated with armed violence and the coercion exercised by non-State armed groups, which influence collective decision-making and electoral processes through the use of force, thereby restricting the effective exercise of political rights. In contexts of constant threat, stigmatization of community leaders, and territorial surveillance, expressing opinions, organizing, or questioning decisions is perceived as high-risk behavior. This climate of fear operates as a mechanism of social control that weakens democratic deliberation:

E6: “*Uno ya no puede elegir gobernante, en muchos territorios uno vota por quienes toca votar ¿si me entiende?*” [You can no longer choose who governs; in many territories, you vote for whoever you are told to vote for, do you understand?].

In addition, the absence and persistent weakness of the State in these territories has facilitated the consolidation of parallel forms of governance, through which illegal armed groups assume functions of social regulation, territorial control, and selective provision of basic goods and services. These dynamics not only fill institutional voids but also reconfigure local power relations:

E24: “*Esos grupos les ha construido a las comunidades casetas comunales...*” [Those groups have built community halls for the communities...].

The construction of community infrastructure by non-State armed groups reinforces their territorial legitimacy and generates relationships of dependency with communities, in which access to goods and services becomes conditioned by implicit loyalties and imposed norms. As a result, formal political participation is weakened and trust in democratic institutions is eroded.

From an SDH perspective, this political configuration shapes the unequal distribution of power and resources, restricting communities’ capacity to influence decisions that directly affect their living conditions, access to basic services, and health protection. In this context, political violence acts as a structural determinant that conditions both opportunities for well-being and the collective capacity to organize, claim, and exercise rights.

The results further show that violence has direct effects on environmental determinants of health. Contamination of water sources due to illicit activities, aerial fumigation, and natural resource exploitation, alongside the loss of food sovereignty, significantly alter the ecosystems upon which these communities depend:

E4: “*Hoy tenemos muchísima contaminación de las fuentes hídricas con químicos por laboratorios y también por explotación de hidrocarburos*” [Nowaday, water sources are deeply contaminated with chemicals from laboratories and also from hydrocarbon extraction].

E22: “*Entonces con el tema de la fumigación, el pan coger desapareció totalmente*” [With the fumigation, subsistence crops disappeared completely].

E30: “*Usted mira el bosque aquí no mira planta mala... decimos ese médico ya no cura porque la planta está envenenada*” [When you look at the forest here, you no longer see medicinal plants... we say that this medicine no longer heals because the plant is poisoned].

Environmental degradation, in addition to compromising nutritional security, also fractures ancestral relationships with territory and medicinal plants, thereby weakening traditional systems of care and healing. These changes increase exposure to disease, reduce autonomy in health management, and deepen dependence on formal healthcare systems, to which access paradoxically remains limited.

Regarding access to health services, the findings reveal structural barriers associated with geographies of fear, territorial control, institutional discrimination, and persistent shortages in infrastructure, personnel, and medical specialties. Restricted mobility, fear of retaliation, and discriminatory treatment, particularly affecting LGBTQ+ populations, reduce care-seeking behaviors and delay timely diagnosis and treatment:

E11: “*La zona rural es más compleja... gastan mucha plata o a veces arriesgan la vida... tratan de solucionar las cosas con los curanderos o parteras*” [The rural area is more complex... people spend a lot of money or sometimes risk their lives... they try to resolve health issues with healers or midwives].

E21: “*No hay acceso a una salud de calidad... no hay garantía de una atención con especialistas permanentes...*” [There is no access to quality healthcare... there is no guarantee of care from permanent specialists...].

These barriers deepen existing health inequities and increase the risk of complications, avoidable deaths, and mental health deterioration, particularly among people facing greater economic vulnerability, LGBTQ+ populations, and dispersed rural communities, whether terrestrial or fluvial, where transport is limited and entails high costs.

The findings further show that rights associated with public services such as health, education, electricity, drinking water, basic sanitation, housing, communication, and transport, are systematically and differentially affected. Disruptions to fluvial and land transport limit the supply of medical inputs, food, and fuel; hinder regular school attendance; restrict maintenance of electricity and water networks; and obstruct the arrival of technical and administrative personnel to the territories. This lack of service coverage weakens local institutional capacity to respond to population needs and reinforces scenarios of structural exclusion and dependence on informal or provisional solutions:

E1: “*Estos territorios ya tienen injerencia en grupos armados, entonces ahí sí ya pues no estaríamos ni en la capacidad de cubrir todas las necesidades que se presentan*” [These territories are already under the influence of armed groups, so we are no longer even able to meet all the needs that arise].

Violence thus shapes the economic, sociocultural, political, environmental, and health conditions under which the life trajectories of Indigenous and Afro-descendant communities unfold, including armed governance, illicit economies, normalized fear, and limited access to basic services (health, education, electricity, drinking water, basic sanitation, housing, communication, and transport). These conditions do not operate in isolation, but rather articulate an interconnected set of interdependent determinants that erode community capacities and deepen historical inequalities. From this perspective, violence, beyond causing direct harm, structurally restricts opportunities for well-being, producing differentiated material, physical, and emotional impacts according to gender, life course, and ethnic belonging in these territories.

## Discussion

From the perspective of the SDH framework, the findings indicate that in the 11 municipalities in the Nariño Pacific region studied, violence operates as a structural determinant of health for Indigenous and Afro-descendant communities. As Farmer ^1^ explained, this dynamic operates through direct expressions of violence that consolidate the governance and dominance of armed groups who, through restrictions, an economy of fear, and the law of silence, drive these communities toward the disconnection of social and institutional cohesion networks (including health services), labor informality, impoverishment, and dependence on illicit economies. This context fosters increased mortality, chronic physical and mental illnesses, the disruption of referral and counter-referral systems, the desertion of health personnel, the intermittent closure of institutional services, and the loss of ancestral knowledge associated with protection and care networks (E3, E10, E13, E18, E50, E60) ^22^ ^,^ ^23^.

As noted by ACAPS ^17^, macro-power and micro-power dynamics can be identified in the way armed groups operate, generating naturalized mechanisms of subjugation among individuals and social groups within these territories. This reveals a scenario that may be associated with the history of slavery experienced by Indigenous and Afro-descendant peoples, in the presence of a circular dynamic of violence that continues to repeat itself within these communities and must be prioritized ^24^ ^,^ ^25^ ^,^ ^26^. In this context, narratives emerge that attribute power and social status to armed groups, accompanied by the normalization of risk, chronic fear, and the internalization of norms and symbols that shape thoughts, emotions, and behaviors associated with the perpetuation of violence (E7, E12, E14, E23, E28, E31) ^27^ ^,^ ^28^.

The coercive action exerted by the dominant power of armed groups, aimed at sustaining the system of ideas and symbols that perpetuate violence ^7^ ^,^ ^29^, is implicated in the construction of scenarios of economic deprivation and structural inequality in these territories. In such contexts, access to basic rights - such as health, education, employment, and public services - for ethnic populations is restricted, becoming a breeding ground for experiences that increase the risk of individuals becoming either victims or perpetrators of violence. This occurs as practices based on illegality emerge at the family, community, and institutional levels as means of survival (E1, E6, E10, E13, E19, E29, E54, E57) ^12^. In this regard, as described by Taussig ^30^, the symbolic and semantic burden encapsulated in the phenomenon of violence through domination in these ethnic territories becomes evident. Likewise, violence is recognized as a direct manifestation of structural inequalities, where determinants such as social inequality and inequity - and intermediate determinants represented by the material conditions of social vulnerability in which the lives of individuals, families, and communities unfold - are associated as precursors and generators of violence ^7^.

In coherence with Krieger’s theoretical framework ^31^, the approach to the SDH must consider the structural, systematic, and semantic disposition of violence within sociocultural, economic, political, environmental, and health service contexts. In these settings, poverty, lack of educational and employment opportunities, institutional negligence and inefficacy, barriers to accessing health services, residential segregation, and the absence of basic infrastructure create living conditions that exacerbate social tensions and fuel conflicts within families and communities as responses to perceived structural oppression (E3, E5, E6, E8, E17, E20, E25) ^32^ ^,^ ^33^ ^,^ ^34^.

Therefore, addressing violence against Indigenous and Afro-descendant communities of the Colombian Pacific from a public health perspective requires recognizing it as a symptom of failed policies and structural inequalities. Marmot ^10^ suggests that violence does not occur in isolation nor as the exclusive consequence of individual factors; rather, he recognizes that social inequities - including access to basic resources and the quality of health services - increase the risks of violence and social conflict. Consequently, public policies must focus on reducing social inequalities to improve health and prevent violence ^35^. This includes a fairer distribution of resources, access to employment, improved working conditions, and dignified living standards, as well as the promotion of equity in access to healthcare and the strengthening of the quality, reach, and effectiveness of social and health services at both individual and collective levels, all under a comprehensive and inclusive ethnic approach ^32^.

## Conclusion

Afro-descendant and Indigenous communities in the Nariño Pacific region are being physically and psychologically affected by multidimensional violence, manifested through direct violence (an extortionist and coercive regime marked by threats, attacks, and confinements that undermine coexistence, access to healthcare, basic public services, and the local economy); cultural violence (the imposition of normative patterns foreign to their culture that disrupt self-governance and ancestral care systems); and structural violence (the parallel governance of armed groups that has weakened institutional structures and formal economies, reorganizing political patterns, markets, and cultural practices, thereby restricting access to health, education, and employment).

A structural, systemic, and symbolic disposition of violence is identified within the social determinants of health (sociocultural, economic, political, environmental, health services, road infrastructure, and basic public services). This configuration is sustained through symbols of domination perpetuated throughout history in these ethnic territories and reinforced by persistent institutional crises, limited access to basic rights, mistrust toward the State, corruption, and the control exerted by armed groups.

When understood through the framework of the SDH, violence reveals that Afro-descendant and Indigenous communities in the Nariño Pacific region are born, grow, work, and age within a context that exposes them to conditions increasing the risk of becoming both victims and perpetrators of violence.

The findings show that, according to life course and gender, there is a greater propensity for victimization among children (risk of recruitment and trauma), adolescents and youth (co-optation and criminalization), women (sexual and economic violence), and men (selective persecution, extortion payments, and homicide), within contexts marked by invisible borders and violations based on ethnicity, social class, and sexual orientation.

Several limitations emerged during data collection, primarily related to mobility restrictions caused by road closures and deteriorating security conditions in certain territories due to clashes between armed groups operating outside the law. Security concerns also influenced the willingness of some participants to disclose information related to violence. It is therefore recommended that future research in contexts of armed conflict carefully design protective measures that ensure the safety of both participants and researchers, in close collaboration with the communities involved in the study.

## Data Availability

The research data are available upon request to the corresponding author.
